# Increased Difficulties in Managing Stairs in Visually Impaired Older Adults: A Community-Based Survey

**DOI:** 10.1371/journal.pone.0142516

**Published:** 2015-11-06

**Authors:** Chen-Wei Pan, Hu Liu, Hong-Peng Sun, Yong Xu

**Affiliations:** 1 Jiangsu Key Laboratory of Preventive and Translational Medicine for Geriatric Diseases, School of Public Health, Medical College of Soochow University, Suzhou, China; 2 Department of Ophthalmology, the First Affiliated Hospital of Nanjing Medical University, Nanjing, China; Zhongshan Ophthalmic Center, CHINA

## Abstract

**Purpose:**

Managing stairs is a challenging aspect of daily activities of living for older people. We assessed whether older adults with visual impairment (VI) have greater difficulties of managing stairs in daily lives.

**Methods:**

The study was designed as a community-based cross-sectional study based on a Chinese cohort aged 60 years and older in rural China. Visual acuity (VA) was measured in both eyes using a retro-illuminated Snellen chart with tumbling-E optotypes. VI (including blindness) was defined as presenting VA of worse than 20/60 in either eye. Having any difficulties in managing stairs was self-reported based on a question drawn from the Barthel Index. Information on participants’ socioeconomic status, lifestyle-related factors, diseases histories and medication intake was collected using a questionnaire.

**Results:**

The Barthel Index, Activities of Daily Living questionnaire was completed by 4597 (99.7%) participants including 2218 men and 2379 women. The age of the participants ranged from 60 to 93 years with a mean of 67.6 ± 6.3 years. In age and gender adjusted models, adults with VI had a higher likelihood of having difficulties in managing stairs (odds ratio [OR] = 2.7; 95% confidence interval [CI] 2.0, 3.7) compared with those without. The association of VI with the likelihood of having difficulties in managing stairs was stronger in older adults who lived alone (OR = 3.2; 95%CI 1.8, 4.5) compared with those who lived with other family members (OR = 2.0; 95%CI 1.3, 4.3). Compared with hypertension, diabetes, obesity and cognitive dysfunction, VI had the greatest impact on people's abilities of managing stairs.

**Conclusion:**

VI was associated with an increased likelihood of having difficulties in managing stairs, especially in those who lived alone. However, whether the finding could be extrapolated to other populations warrants further studies as different environmental exposures such as illumination and types of stairs may alter the association observed in this study.

## Introduction

Visual impairment (VI) is one of the most devastating disabilities throughout the world. It was estimated that 285 million people are visually impaired worldwide.[1] The absolute number of visually impaired people is still increasing, which is driven by population aging, longer life expectancy and increasing burden of chronic systemic diseases such as diabetes and hypertension.[2] Although nowadays vision screening outreach and eye care programs have become increasingly widespread in both developed and developing countries, quite a few people with VI remain undiagnosed and untreated.[3] VI is intimately associated with functional limitations[4], falls[5], depressive symptoms[6], cognitive dysfunction[7], worse health-related quality of life[8] and increased risk of mortality[9, 10]. Visual impairment and its consequence are responsible for consuming a huge share of healthcare costs and impose heavy socioeconomic burden on the individuals, communities and countries. It was reported that VI costs the world an estimated 2.3 trillion dollars annually.[3] In mainland of China, the prevalence of VI varies significantly by geographic regions. The prevalence of VI was estimated to be 3.6% in East China, 3.6% in Central China and 5.2% in Western China while the prevalence of blindness was 1.4% in East China, and 1.4% in Central China and 2.5% in Western China.[11]

VI has been linked with restricted daily activities. Managing stairs is a challenging aspect of daily activities of living among older adults. It was reported that climbing up and down stairs has been rated as one of the top five tasks which are difficult to perform for older people in daily lives.[12] Literature from developed countries has shown that older people with chronic conditions and without partners have greater difficulties in managing stairs.[13] The impact of VI on people’s ability of managing stairs remains unclear and needs to be assessed.

In this study, we assessed the impact of VI on the likelihood of having difficulties in managing stairs in a group of older Chinese people living in a rural community. In addition, we also compared the impact of VI on the ability of managing stairs with that of other common comorbidities among the older adults including hypertension, diabetes, obesity and cognitive dysfunction in this cohort.

## Materials and Methods

### Study population

The Weitang Geriatric Diseases study was a community-based survey conducted in the Weitang town located in Suzhou in eastern China in 2014. The aim of the study was to estimate the patterns, predictors and burden of common health outcomes of older people aged 60 years or older in this area. Based on official records, 6,030 people aged 60 years and older resided in the town. Before the study, an invitation letter was sent to each family and the nature of the study was explained in the letter. All the adults aged 60 years or older in the town were invited to participate in this study. An adult was considered “ineligible” to participate in this study if he or she had moved from the residing address, had not been living there for more than 6 months, or was deceased. Of the 6,030 names listed in the official records, 5,613 subjects were considered to be “eligible” to participate in this study. In the end of the study, 4,611 older adults (82.1%) participated in this study. Compared with non-responders, responders of this study were younger (P = 0.01) but there were no gender differences (P = 0.52).

The Weitang Geriatric Diseases study was conducted following the tenets of the Helsinki Declaration and was approved by the Institutional Review Board of Soochow University. All participants gave written informed consent at the recruitment stage of the study.

### Measurement of difficulties in managing stairs

Having any difficulties in managing stairs was self-reported based on a question in the Barthel Index, Activities of Daily Living (ADL)[14, 15] which asked “do you need help in climbing stairs”. Responses for this question included “unable”, “need help” or “independent”. Adults whose answer was “unable” or “need help” were categorized as “having difficulties in managing stairs” while those whose answer was “independent” were defined as “having no difficulties in managing stairs”.

### Measurement of visual impairment

Participants underwent comprehensive eye examinations including auto-refraction, slit-lamp examination and fundus photography. Visual acuity (VA) was measured in both eyes by trained research optometrists using a retro-illuminated Snellen chart with tumbling-E optotypes (Precision Vision, La Salle, IL, USA) at a distance of 4 m. A line was regarded as completed with at least 4 out of 5 optotypes were identified clearly. The presenting visual acuity (PVA) was recorded with the participant wearing his or her habitual optical correction such as spectacles or contact lenses, if any. Best-corrected visual acuity was measured after correcting any refractive errors. If no number on the chart could be read at 4 m, the participant was moved to 3, 2, or 1 m, consecutively. If no number could be read at all, VA was examined as counting fingers, hand movements, perception of light, or no perception of light. VI (including blindness) was defined as PVA of worse than 20/60 in either eye. This definition was different from the World Health Organization definition.

### Assessment of covariates

A risk factor questionnaire collecting information regarding study participants’ socioeconomic status (e.g. education, occupation, monthly income), lifestyle-related factors (e.g. smoking, alcohol intake, tea consumption, sleeping hours), diseases histories and medication intake was administered by trained research assistants. Each participant’s height was measured in centimeters using a wall-mounted measuring tape, after removing shoes while weight was measured in kilograms using a digital scale, after removing heavy clothing. Systolic, diastolic blood pressure and pulse rate were measured using an automated blood pressure monitor, following the protocol used in the Multi-Ethnic Study of Atherosclerosis.[16] Body mass index (BMI) was calculated as the weight in kilograms divided by the square of the height in meters and obesity was defined as BMI of ≥28 kg/m^2^ (Chinese adult population standard).[17] Diabetes mellitus was defined as fasting glucose levels of more than 7.0 mmol/L or physician diagnosis of diabetes and use of diabetic medications.[18] Hypertension was defined as systolic blood pressure of 140mmHg or more or diastolic blood pressure of 90mmHg or more, physician diagnosis of hypertension, or use of antihypertensive medications.[19] The Abbreviated Mental Test (AMT) was used to assess participants’ general cognitive function including orientation, semantic knowledge, episodic memory, delayed recall, picture naming, and attention. The presence of cognitive dysfunction was defined as an AMT score of 6 or less out of 10 for those with 0 to 6 years of formal education and an AMT score of 8 or less out of 10 for those with more than 6 years of formal education.[20]

### Statistical analysis

Binary logistic regression models were established to estimate the associations between VI including both unilateral and bilateral VI and the presence of having any difficulties in managing stairs. The effect estimate of odds ratio (OR) and its relative 95% confidence interval (CI) were calculated. For multivariate analysis, three models were constructed. Model 1 was the reference model which adjusted for age and gender only. Model 2 and 3 additionally adjusted for other potential confounders and were grouped as follows: Model 2: age, gender and socioeconomic status (education, income, and living alone or not); Model 3: variables in Model 2 plus lifestyle-related exposures (smoking, alcohol intake, tea consumption, and sleeping hours per day). Interaction effects (different combination of the variables) between VI and other risk factors on the likelihood of having any difficulties in managing stairs were determined using a likelihood ratio test. The effect of other comorbidities such as hypertension, diabetes, obesity and cognitive dysfunction were analyzed in the same way and the effect estimates of these comorbidities were compared with that of VI. All probabilities quoted were considered statistically significant if a P value was less than 0.05 and all data analyses were performed using SPSS (PASW Statistics 18, SPSS Inc, Chicago, IL).

## Results

Of the 4,611 older adults who participated in this study, the Barthel Index, Activities of Daily Living questionnaire was completed by 4,597 (99.7%) participants. Among the 4,597 participants, 4,351 (95.0%) reported that they could manage stairs independently, 205 (4.5%) reported that they need help when managing stairs while only 23 (0.5%) responded that they could not manage stairs. Therefore, 228 adults were categorized as “having difficulties in managing stairs” and the other 4,351 were categorized as “having no difficulties in managing stairs”. Table 1 summarized the characteristics of the study participants with and without any difficulties in managing stairs. In this study, older adults with any difficulties in managing stairs were more likely to be older (P<0.001), female (P = 0.001), have lower AMT scores (P<0.001), live alone (P<0.001) and have higher prevalence of hypertension (P = 0.001) and diabetes (P = 0.06) compared with those without. Meanwhile, they had less monthly income (P<0.001), were less educated (P<0.001), were less likely to smoke (P<0.001), drink alcohol (P<0.001) or tea (P<0.001), and slept for more hours per day (P<0.001).

**Table 1:** Characteristics of people with and without difficulties in managing stairs.

	With difficulties in managing stairs (n = 228)	Without difficulties in managing stairs (n = 4351)	P value*
Age (years)	74.0 (7.6)	67.3 (6.1)	<0.001
Female gender	144 (63.2)	2235 (51.4)	0.001
Individual monthly income less than 1000 Yuan	185 (81.1)	2437 (56.0)	<0.001
No formal education	151 (66.2)	2035 (46.8)	<0.001
Living alone	41 (18.0)	378 (8.7)	<0.001
Obesity	23 (10.1)	594 (13.7)	0.02
Hypertension	157 (68.9)	2618 (60.2)	0.001
Diabetes	24 (10.5)	313 (7.2)	0.06
Abbreviated Mental Test scores	7.8 (1.9)	8.7 (1.6)	<0.001
Ever smoked	48 (21.1)	1524 (35.0)	<0.001
Alcohol intake within the past 3 month	25 (11.0)	1010 (23.2)	<0.001
Drinking tea for at least one year	53 (23.2)	1518 (34.9)	<0.001
Sleeping hours per day (hours)	9.5 (1.6)	8.7 (1.4)	<0.001

Data presented are means (standard deviations) or number (%), as appropriate for variable.

*P value, comparing the differences between people with and without difficulties in managing stairs, based on chi-square test or t test, as appropriate.

Table 2 demonstrates the association of VI with the likelihood of having any difficulties in managing stairs in this cohort. In age and gender adjusted models, adults with VI had a higher likelihood of having difficulties in managing stairs (OR = 2.7; 95% CI 2.0, 3.7) compared with those without (Model 1). The magnitude of association reduced when other potential confounders including socioeconomic status and lifestyle-related exposures were additionally adjusted but remained statistically significant (Model 2 and 3). The associations were also significant when bilateral VI (VI in both eyes) and unilateral VI (VI only in one eye) was analyzed separately. Although people with bilateral VI seem to have greater difficulties in managing stairs compared those with unilateral VI, the difference did not reach statistical significance (P>0.05).

**Table 2:** Relationship of visual impairment (VI) with the likelihood of having difficulties in managing stairs.

	Model 1			Model 2			Model 3		
	OR	95%CI	P	OR	95%CI	P	OR	95%CI	P
With VI vs. without VI	2.7	2.0,3.7	<0.001	2.3	1.5,3.2	<0.001	2.2	1.4,3.3	<0.001
With unilateral VI vs. without VI	2.4	1.6,3.4	<0.001	1.9	1.2, 3.0	0.003	1.9	1.2, 3.2	0.01
With bilateral VI vs. without VI	3.5	2.3,5.3	<0.001	3.2	2.0, 5.2	<0.001	2.9	1.7, 5.0	<0.001
With bilateral VI vs. with unilateral VI	1.5	0.9,2.5	0.11	1.3	0.8, 2.4	0.35	1.2	0.7, 2.6	0.38

OR = odds ratio; CI = confidence interval. Factors adjusted for in each model: Model 1: age and gender; Model 2: age, gender, educational level; monthly income, and living alone or not; Model 3: age, gender, educational level; monthly income, living alone or not, smoking, alcohol intake, tea consumption, and sleeping hours per day

A significant joint effect of living arrangement with VI on the likelihood of having any difficulties in managing chairs was detected using a likelihood ratio test (P for interaction = 0.04). Further stratified analysis indicated the association of VI with the likelihood of having difficulties in managing stairs was stronger in older adults who lived alone (OR = 3.2; 95%CI 1.8, 4.5) compared with those who lived with other family members such as partners or kids (OR = 2.0; 95%CI 1.3, 4.3) in multivariate analysis.

We also compared the effect estimates of five common comorbidities including VI, hypertension, diabetes, obesity and cognitive dysfunction on the likelihood of having difficulties in managing stairs in this cohort. To facilitate comparison, we adjusted age and gender only in all the models. The OR was 1.5 (95% CI 1.1, 2.5) for hypertension, 1.1 (95% CI 0.95, 2.9) for diabetes, 1.2 (95% 0.85, 3.3) for obesity, and 2.2 (95% CI 1.2, 4.6) for cognitive dysfunction, respectively. Thus, VI had the greatest impact on people’s abilities of managing stairs among the five common comorbidities in older adults.

## Discussion

In this community-based survey of older Chinese aged 60 year or older, we reported that VI was associated with an increased likelihood of having difficulties in managing stairs and this association was independent of age, gender, socioeconomic status and lifestyle-related parameters. The magnitude of association was even stronger in those who lived alone compared with those who lived with family members. Compared with other common chronic comorbidities, VI had a greater impact on people’s ability of managing stairs. Our study indicated that proper treatment on VI and counselling on visual rehabilitation should be integrated into health care services for older adults.

Managing stairs is a common activity in daily lives, especially in rural areas where electronic elevators are not always available. Compared with mobility on level surface, managing stairs is much more challenging for older people. Fall on stairs is a common cause of injuries and death among older adults. Previous studies have shown that VI could significantly increase the risk of falls[5, 21–23] and was positively associated with the risk fracture[23, 24] among older adults. Although there is clear evidence suggesting that older adults with VI have a higher risk of fall, only a limited number of studies have investigated the difficulties of managing stairs in elder adults associated with visual impairment. To the best of our knowledge, only one study conducted in Malaysia have focused on this topic (n = 907).[25] Our study had a much larger sample size and highlighted the importance of vision when managing stairs among older adults living alone and those living with others. Dysfunction in vision could have a directly impact on the ability of managing stairs among older adults. This is simply because persons with VI could not see clearly when climbing up and down stairs. On the other way, vision-threatening eye diseases such as cataract may reflect systemic health and ageing of the whole body[10], which may be associated with a greater likelihood of reporting mobility difficulty and greater risk of developing mobility disability. In the stratified analysis, the influence of VI on visual impairment varied with living arrangements. More specifically, the detrimental effect of VI was stronger in people living alone. A possible explanation for this interaction effect may be that individuals who live alone had experienced greater difficulties and economic pressure that kept them away from general health and eye care services, and ultimately they were more vulnerable to VI compared with the ones living with other family members.

Some important public health implications of our study should be noted. Disability of managing stairs among older adults may result in fracture and increase the risk of mortality. With rapid progress of urbanization in China during the past few decades, many younger people migrated from rural countries to unban cities to make a living, leaving their parents alone at home. Our study now showed that many of these visually impaired older people who live alone were unable to manage stairs on their own. Efforts and resources could be channeled towards the correction of VI such as cataract surgery and vision rehabilitation such as counselling and low vision clinical services. Low-cost, safe, and easy-to-operate approaches targeting the prevention of VI are also needed. In addition, VI results from age-related eye diseases such as aged-related cataract, age-related macular degeneration, diabetic retinopathy and glaucoma.[26–33] Considering that these vision-threatening eye diseases are closely linked with systematic chronic diseases such as hypertension and diabetes, controlling blood pressure and glucose in older people living alone should not be neglected.

Although this study had several strengths, including a large and representative sample and the using of a standardized protocol to measure VA, there were still some limitations, which need to be acknowledged. The determination of whether people had difficulties in managing chairs relied on a simple question drawn from the Barthel Index. Polite studies were not performed and its validity in evaluating managing stairs using one single question from the Barthel Index remains uncertain. In practice, stairs may differ in their designs and are present in different environments. Such detailed information was not captured in our studies. In addition, although we had adjusted for a wide range of confounders including socioeconomic variables and lifestyle-related factors in multivariate analyses, residual confounding may still exist, which might have biased the results. For example, some important risk factors related to managing stairs including mobility and history of stroke were not captured in this study and thus were not included in the analysis. Furthermore, we only assessed the impact of VI on the difficulties in managing stairs. Other visual dysfunctions such as contrast sensitivity, glare, and visual field defect were not considered as these functions were not measured in this study. Finally, we cannot determine whether VI predates the occurrence of having difficulties in managing stairs or to what extent self-reported difficulties in managing stairs may affect VI diagnosis as measured subjectively considering the cross-sectional nature of the study.

In summary, this community-based study of people aged 60 years or older indicated that visually impaired people had greater difficulties in managing stairs compared with those without VI. Our study would be helpful for planning an appropriate and effective vision rehabilitation program for older people aiming at reducing their risk of falling on stairs and increasing their independence level in daily lives. However, whether the finding could be extrapolated to other population warrants further studies as other environmental exposures such as illumination and types of stairs many alter the association observed in this study.
